# Supplementary figures and images for: Ward-level factors associated with methicillin-resistant Staphylococcus aureus acquisition–an electronic medical records study in Singapore
Source: PLoS One. 2021 Jul 22;16(7):e0254852. doi: 10.1371/journal.pone.0254852 (PMC8297767; doi:10.1371/journal.pone.0254852)

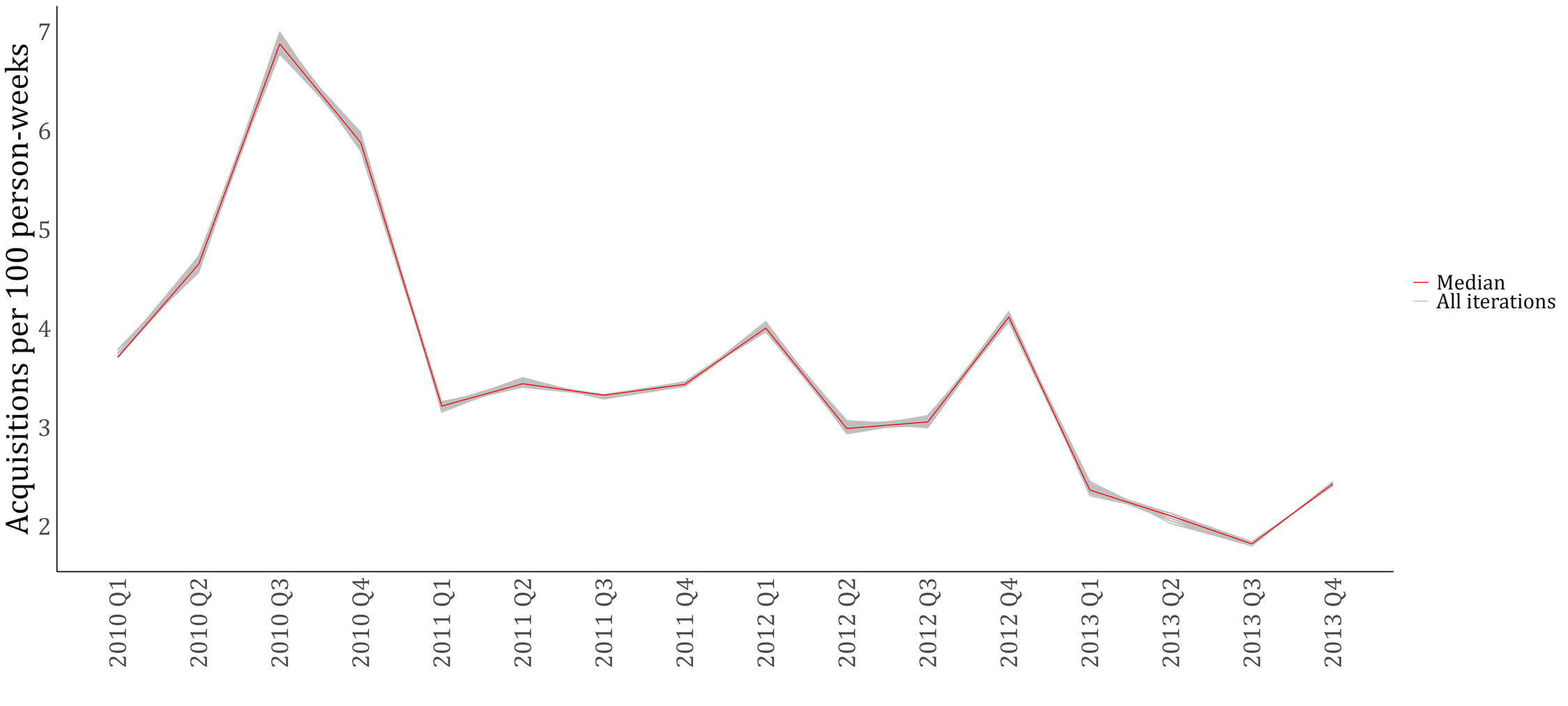

Supplement: S1 Fig — (TIF) [file pone.0254852.s001.tif]
