# Supplementary material for: Ward-level factors associated with methicillin-resistant Staphylococcus aureus acquisition–an electronic medical records study in Singapore
Source: PLoS One. 2021 Jul 22;16(7):e0254852. doi: 10.1371/journal.pone.0254852 (PMC8297767; doi:10.1371/journal.pone.0254852)
Supplement: S1 Table — (DOCX) [file pone.0254852.s002.docx]

## S2 Table. MRSA acquisition rate in MRSA active screening wards of National University Hospital, 2010-2013

| **Ward^** | **Acquisition rate*** | **95% CI** | **Median MRSA acquisitions^#^** | **Median person-week at risk^#^** |
| --- | --- | --- | --- | --- |
| 11 Surgery, HDU | 12.7 | 4.1, 29.7 | 5 | 39 |
| 6 Medical, GRM | 6.2 | 5.4, 7.0 | 235 | 3,811 |
| 2 Orthopaedics | 5.7 | 4.9, 6.6 | 194 | 3,394 |
| 3 Cardiac | 4.9 | 4.3, 5.6 | 259 | 5,267 |
| 4 Cardiac | 4.9 | 4.2, 5.6 | 201 | 4,118 |
| 2 Medical | 4.5 | 3.9, 5.1 | 194 | 4,354 |
| 5 Medical, Renal | 4.5 | 3.9, 5.2 | 197 | 4,383 |
| 8 Medical, Neuro | 4.4 | 3.5, 5.4 | 80 | 1,834 |
| 4 Surgery | 4.3 | 3.7, 5.0 | 172 | 3,960 |
| 2 Cardio-Thoracic ICU | 4.0 | 3.0, 5.3 | 51 | 1,271 |
| 5 Surgery | 3.9 | 3.3, 4.5 | 172 | 4,415 |
| 1 Medical, ICU, HDU | 3.6 | 2.7, 4.6 | 62 | 1,742 |
| 1 Surgery, HDU | 3.6 | 2.8, 4.6 | 66 | 1,816 |
| 3 Surgery, ICU | 3.5 | 2.3, 4.9 | 31 | 897 |
| 2 Neurosurgery, HDU | 3.4 | 2.3, 4.8 | 32 | 949 |
| 1 Cardiac | 3.2 | 2.2, 4.5 | 34 | 1,053 |
| 3 Other ward | 3.0 | 0.4, 10.7 | 2 | 67 |
| 2 Oncology | 2.9 | 2.3, 3.7 | 72 | 2,474 |
| 1 Oncology, Medical | 2.9 | 2.1, 3.9 | 43 | 1,485 |
| 6 Surgery | 2.8 | 2.2, 3.5 | 73 | 2,613 |
| 5 Coronary Care | 2.7 | 1.5, 4.5 | 14 | 526 |
| 2 Isolation | 2.6 | 1.9, 3.4 | 49 | 1,912 |
| 4 Oncology | 2.5 | 2.0, 3.3 | 64 | 2,513 |
| Medical, Surgery mixed | 2.5 | 1.8, 3.3 | 46 | 1,833 |
| 9 Surgery | 2.2 | 1.4, 3.2 | 24 | 1,108 |
| 3 Oncology | 1.9 | 1.4, 2.4 | 54 | 2,903 |
| 1 Orthopaedics | 1.7 | 1.3, 2.2 | 61 | 3,556 |
| 9 Medical | 1.5 | 0.8, 2.4 | 15 | 1,031 |
| 4 Medical, HDU | 1.4 | 0.4, 3.2 | 5 | 364 |
| 3 Orthopaedics | 1.0 | 0.4, 2.1 | 6 | 620 |
| 6 Oncology, Medical | 1.0 | 0.5, 2.0 | 9 | 874 |
| 8 Surgery | 1.0 | 0.5, 1.9 | 10 | 960 |
| 10 Surgery | 0.7 | 0.2, 1.9 | 4 | 548 |
| 8 Oncology | 0.5 | 0.2, 1.2 | 5 | 964 |
| 5 Oncology, HDU | 0.4 | 0.1, 0.9 | 4 | 1,095 |
| 7 Oncology | 0.4 | 0.1, 1.0 | 4 | 1,029 |
| CI, Confidence interval; HDU, High dependency unit; ICU, Intensive care unit | | | | |
| * Number of MRSA acquisitions per 100 person-weeks | | | | |
| ^ The ward numbers are masked | | | | |
| # Median value from 10,000 iterations in the main analysis | | | | |
