# Supplementary material for: Ward-level factors associated with methicillin-resistant Staphylococcus aureus acquisition–an electronic medical records study in Singapore
Source: PLoS One. 2021 Jul 22;16(7):e0254852. doi: 10.1371/journal.pone.0254852 (PMC8297767; doi:10.1371/journal.pone.0254852)
Supplement: S2 Table — (DOCX) [file pone.0254852.s003.docx]

## S3 Table. Results of main analysis before imputing screening results for subsequent hospitalisations of a known MRSA case

| **Ward characteristics** | **Unadjusted RR**  **(95% CI)** | **Adjusted RR**  **(95% CI)** |  |
| --- | --- | --- | --- |
| Critical care ward |  |  |  |
| No | 1 | 1 |  |
| Yes | 1.09 (0.66, 1.80) | 1.74 (1.10, 2.74) |  |
| Presence of MRSA cohorting beds |  |  |  |
| No | 1 | 1 |  |
| Yes | 1.52 (0.99, 2.34) | 1.34 (0.96, 1.88) |  |
| Ward specialty |  |  |  |
| Medical | 1 | 1 | |
| Oncology | 0.37 (0.23, 0.60) | 0.61 (0.41, 0.92) |  |
| Ortho | 1.19 (0.63, 2.22) | 0.80 (0.51, 1.27) |  |
| Other | 1.01 (0.67, 1.54) | 1.21 (0.81, 1.81) |  |
| Surgery | 0.99 (0.65, 1.51) | 0.87 (0.62, 1.22) |  |
| MRSA prevalence among directly admitted patients (additional 2 percentage point) | 1.66 (1.41, 1.95) | 1.50 (1.27, 1.78) |  |
| MRSA prevalence among patients transferred from other wards (one additional 6 percentage point) | 2.56 (1.81, 3.62) | 2.97 (1.95, 4.52) |  |
| Number of patients on a typical day^ (18 additional patients) | 1.39 (1.11, 1.76) | 1.20 (0.95, 1.52) |  |
| Median length of stay (1.5 additional days) | 0.75 (0.59, 0.95) | 0.68 (0.52, 0.87) |  |
| Indegree (one additional ward) | 1.82 (1.02, 3.24) | 0.99 (0.55, 1.78) |  |
| Weighted-indegree (101 additional patients) | 5.38 (1.45, 19.90) | 2.60 (0.72, 9.39) |  |
| Interaction term* |  | 1.11 (1.02, 1.22) |  |

CI, Confidence interval; RR, rate ratio

^ Proxy for ward patient capacity

* Interaction of MRSA prevalence among transfer patients and weighted in-degree
