# Supplementary material for: Ward-level factors associated with methicillin-resistant Staphylococcus aureus acquisition–an electronic medical records study in Singapore
Source: PLoS One. 2021 Jul 22;16(7):e0254852. doi: 10.1371/journal.pone.0254852 (PMC8297767; doi:10.1371/journal.pone.0254852)
Supplement: S3 Table — (DOCX) [file pone.0254852.s004.docx]

## S4 Table. Comparing model results with and without hand hygiene compliance

| **Ward characteristics** | **Models including hand hygiene compliance** | | **Main analysis** |
| --- | --- | --- | --- |
|  | **Unadjusted RR^$^ (95% CI)** | **Adjusted RR (95% CI)** | **Adjusted RR (95% CI)** |
| Critical care ward |  |  |  |
| No | 1 | 1 | 1 |
| Yes | 1.33 (0.79, 2.22) | 1.53 (1.09, 2.15) | 1.72 (1.09, 2.70) |
| MRSA cohorting beds |  |  |  |
| No | 1 | 1 | 1 |
| Yes | 1.62 (1.16, 2.26) | 1.59 (1.32, 1.91) | 1.39 (1.03, 1.90) |
| Ward specialty |  |  |  |
| Medical | 1 | 1 | 1 |
| Oncology | 0.51 (0.32, 0.80) | 1.09 (0.87, 1.38) | 0.66 (0.46, 0.94) |
| Ortho | 0.56 (0.32, 0.99) | 0.90 (0.70, 1.16) | 0.81 (0.52, 1.29) |
| Other | 0.82 (0.55, 1.22) | 1.53 (1.23, 1.89) | 1.21 (0.84, 1.76) |
| Surgery | 0.67 (0.45, 1.01) | 1.00 (0.85, 1.17) | 0.91 (0.67, 1.23) |
| MRSA prevalence among directly admitted patients (additional 5 percentage point) | 1.28 (0.91, 1.82) | 0.83 (0.57, 1.21) | 0.75 (0.52, 1.09) |
| MRSA prevalence among patients transferred from other wards (one additional 8 percentage point) | 4.72 (2.81, 7.92) | 7.26 (3.66, 14.40) | 7.74 (3.88, 15.44) |
| Number of patients on a typical day^ (18 additional patients) | 1.46 (1.18, 1.80) | 1.33 (1.07, 1.66) | 1.18 (0.94, 1.50) |
| Median length of stay (1.5 additional days) | 0.84 (0.64, 1.09) | 1.00 (0.80, 1.24) | 0.70 (0.55, 0.90) |
| Indegree (one additional ward) | 2.00 (1.10, 3.62) | 1.13 (0.67, 1.90) | 1.22 (0.69, 2.18) |
| Weighted-indegree (101 additional patients) | 5.59 (1.48, 21.04) | 3.25 (1.45, 7.29) | 2.65 (0.73, 9.68) |
| Interaction term* |  | 1.12 (1.02, 1.22) | 1.11 (1.01, 1.21) |
| Hand hygiene compliance (additional 11 percentage point) | 0.95 (0.91, 1.00) | 0.96 (0.91, 1.01) |  |

CI, Confidence interval; RR, rate ratio

^$^ Wards with absent hand hygiene compliance data were excluded.

^ Proxy for ward patient capacity

* Interaction of MRSA prevalence among transfer patients and weighted in-degree
