# Supplementary material for: Ward-level factors associated with methicillin-resistant Staphylococcus aureus acquisition–an electronic medical records study in Singapore
Source: PLoS One. 2021 Jul 22;16(7):e0254852. doi: 10.1371/journal.pone.0254852 (PMC8297767; doi:10.1371/journal.pone.0254852)
Supplement: S1 File — (ZIP) [file pone.0254852.s005.zip › supplementary/README.docx]

Below is the description of datasets.

| **File name** | **Description** |
| --- | --- |
| mrsa_acquisition_main_analysis.csv | This dataset was used in the main analysis as one of the 10,000 iterations of the main analysis. It includes quarterly MRSA acquisitions, patient week at risk by ward, including other covariates of the multivariable model. |
| fig_2_interaction_plot.csv | This dataset includes estimates used to create the plot in Figure 2. |
| fig_3_sensitivity_analyses_plot_estimates.csv | This includes the estimates used to create the plot in Figure 3. |
